# Physical exercises in the treatment of idiopathic scoliosis at risk of brace treatment – SOSORT consensus paper 2005

**DOI:** 10.1186/1748-7161-1-6

**Published:** 2006-05-11

**Authors:** Hans-Rudolf Weiss, Stefano Negrini, Martha C Hawes, Manuel Rigo, Tomasz Kotwicki, Theodoros B Grivas, Toru Maruyama, members of the SOSORT

**Affiliations:** 1 Asklepios Katharina Schroth Spinal Deformities Rehabilitation Centre, Bad Sobernheim, Germany; 2 ISICO (Italian Scientific Spine Institute), Milan, Italy; 3 University of Arizona, Tucson AZ 85721, USA; 4 Instituto Èlena Salvá, Barcelona, Spain; 5 University of Medical Sciences, Poznan, Poland; 6 Orthopaedic Department "Thriasion" General Hospital, Magula, Athens, Greece; 7 Department of Orthopaedic Surgery, Teikyo University School of Medicine, 2-11-1 Kaga, Itabashi-ku, Tokyo 173-8605, Japan

## Abstract

**Background:**

Based on a recognized need for research to examine the premise that nonsurgical approaches can be used effectively to treat signs and symptoms of scoliosis, a scientific society on scoliosis orthopaedic and rehabilitation treatment (SOSORT) was established in Barcelona in 2004. SOSORT has a primary goal of implementing multidisciplinary research to develop quantitative, objective data to address the role of conservative therapies in the treatment of scoliosis. This international working group of clinicians and scientists specializing in treatment of scoliosis met in Milan, Italy in January 2005.

**Methods:**

As a baseline for developing a consensus for language and goals for proposed multicenter clinical studies, we developed questionnaires to examine current beliefs, before and after the meeting, regarding (1) the aims of physical exercises; (2) standards of treatment; and (3) the impact of such treatment performed by specialists in the field.

**Results:**

The responses to the questionnaires show that, in principle, specialists in scoliosis physiotherapy do not disagree and that several features can be regarded, currently, as standard features in the rehabilitation of scoliosis patients. These features include autocorrection in 3D, training in ADL, stabilizing the corrected posture, and patient education.

## 1. Background

For treatment of all pediatric spinal deformities, the goal is to maintain function and prevent symptoms in the short- and long-term. In children with scoliosis, as summarized below, predictable signs and symptoms including pain and reduced pulmonary function begin early in life and worsen with age. Most curvatures still present at skeletal maturity also continue to worsen throughout life. For children with scoliosis, therefore, optimal treatment goals include reversing curvature magnitude and/or preventing curvature progression, pain, and pulmonary dysfunctionover a lifetime.

### Pain

Most clinical outcome surveys have revealed that, by early adulthood, the majority of scoliosis patients suffer from pain [1-15]. Only one large, controlled survey has been carried out, to date [16]. In that study, 1178 young adults, interviewed 10 years after diagnosis in adolescence, reported a significantly higher incidence of pain than 1217 control subjects. Of the scoliosis patients reporting pain, 23% (147/650) described it as *'horrible, excruciating, distressing' *compared with 1% (6/416) of the control subjects who reported pain. Similar results were reported at >44 year followup [17]. Of a subset of 69 patients treated in adolescence (from an original population of 444), twice as many scoliosis patients (77% vs 35%) suffered from pain compared with a population of adults of comparable age (>55 years). Incidence of chronic pain was almost three fold higher in the scoliosis patients (61%) compared with the controls without scoliosis (22%). This is despite the fact that the 'control' popoulation was selected from hospital clinics, nursing homes, and senior citizens' centers where incidence of disability is exceptionally high [18,19]. How scoliosis causes pain is not clear, but the magnitude of pain in adult scoliosis patients recently has been found to be inversely proportional to curvature flexibility [20]. Related factors linked with pain include regional balance, instability and pathological mechanical loads on spinal elements [21].

### Pulmonary dysfunction

Thoracic scoliosis in children results in characteristic signs of pulmonary dysfunction including reduced vital capacity (VC) and impaired exercise capacity (EC) [22-28]. Because the mechanism for impaired function is reduced mobility of the chest wall and such mobility deteriorates with age, pulmonary function deteriorates according to curvature magnitude even when the curvature itself does not progress [29-33]. In severe cases death occurs by respiratory failure [30-35]. The effects of reduced pulmonary function in patients with mild to moderate scoliosis are not known and have been dismissed as insignificant [e.g., [36,37]]. Recent studies, however, have shown that VC and EC characteristic of patients with mild to moderate scoliosis (<85% predicted) are more reliable predictors of increased mortality than diabetes, high blood pressure, and heart disease [38-40]. Patient-described pulmonary symptoms, in general, are not a reliable indicator because patients usually are unaware of their limitations even when documented signs are severe and respiratory failure is imminent [29-33,41-43].

### Progression

Once a flexible spinal curvature evolves into a spinal deformity, a 'vicious cycle' is initiated in which continuous asymmetric loading of the spinal elements fosters continued progression [44-46]. Only a few small surveys have examined the epidemiology of progression and insufficient information is available to reliably predict outcome for any given patient [47,48]. In general, the danger for dramatic progression is highest during periods of rapid growth, but most cases continue to progress throughout life [1,15,48-50]. Some individuals with similar curves exhibit marked progression after skeletal maturity while others are relatively stable [41]. The bases for such differences are unknown, though some have suggested that the likelihood of progression is greater the more rigid the curvature [51].

### Role of exercise in treatment of scoliosis

Exercise based therapies, alone or in combination with orthopedic approaches, are a logical approach to improve and maintain flexibility and function in patients at risk for pain, pulmonary dysfunction, and progression. Data from the Schroth clinic in Bad Sobernheim, Germany reveal improved pulmonary function [52,53] and reduced pain [54-56] in response to an intensive scoliosis in-patient rehabilitation (SIR) regime. Among the small number of studies which have examined it formally [56-63], progression was less in patient populations who were treated with exercise [reviewed in [64]]. When exercise was prescribed but was not carried out by the patients, progression was similar to untreated populations [60].

The role of exercise based therapies as discussed in the spine literature has been controversial, however, with often-repeated claims that research has shown that such approaches are ineffective in treating scoliosis [e.g. [65-78]]. A systematic review of articles published in English throughout history produced no data in support of such claims [79]. As pointed out by Focarile et al., [80] in 1991, *'Experimental controlled studies of different therapies seem to be justified both on ethical and scientific grounds.'*

SOSORT was established in 2004 to respond to a need for objective scientific information from independent sources. A meeting was held in Milan, Italy, to explore existing community perspectives regarding (1) the aims of physical exercises; (2) standards of treatment; and (3) the impact of such treatment performed by specialists in the field. The goal was to initiate a dialog for building a working consensus prior to initiation of multicenter research initiatives among members of SOSORT.

## 2. Methods

### 2.1. Premeeting-questionaire (before the consensus meeting)

Questionnaires were prepared through consensus among the authors of the study. A first version was drafted by the second author, then critiqued and revised through electronic mail conference to produce a second edition. The second version was submitted to a pre-test by e-mail, to obtain the final form. The title of the questionnaire was "Therapeutic aims of physical exercise treatment in patients at risk of brace treatment."

The following clinical description was given:

*Patient at the start of pubertal growth spurt. Curve at high risk of progression and high risk of prescription of a brace. You propose physical exercises to prevent progression*.

The following questions were asked:

1. What are the **therapeutic aims **of the exercises you propose (i.e what do you want to improve?)

*2. Which aims are more important (****priority****: 1 high – 2 medium – 3 low) ?*

3. **Why **do you choose these aims ?

4. **How **do you obtain these aims ?

During the preparation of the study, the possibly relevant therapeutic aims of the exercises for scoliosis treatment were proposed by the second author and submitted to a preliminary consensus among the authors of this study: The final list was provided in the questionnaire, with space to answer to questions 2, 3 and 4 for each therapeutic aim chosen. The possibly relevant therapeutic aims of the exercises for scoliosis treatment identified by the authors included:

• Autocorrection 3D

• Autoelongation

• Coordination

• Equilibrium

• Ergonomy

• General motor capacity

• Muscular endurance

• Muscular strength

• Neuromotorial control of the spine

• Increase of Range of Motion

• Respiratory capacity

• Respiratory education

• Side-shift

• Stabilisation

Responders could add any relevant aim.

Questionnaires constituted the abstracts of the "SOSORT consensus meeting in Milan, January 2005." These were sent, together with the Preliminary Program, to all the attendees of the "1^st ^International Meeting on Conservative Management of Spinal Deformities" held January 2004 in Barcelona. The program also was distributed to all others with interest in conservative treatment of adolescent idiopathic scoliosis that it was possible to retrieve according to the indexed literature. To gather the maximum possible range of opinions, it was required to fill in the questionnaire independently by the participation to the Consensus Meeting, and to reply by e-mail 1.5 months before the Meeting. 20 persons or institutions responded to the premeeting questionnaire.

### 2.2. Postmeeting-questionnaire (after the consensus meeting)

During the Milano consensus meeting the attendees were asked to fill in the questionnaires after formal discussion.

Thirty attendees took part and filled in the questionnaire to state their opinion about their aims when treating scoliosis patients by physiotherapy. The results can be seen on Table 2.

**Table 1:** Premeeting answers

	Answers	Median	Min	Max
***Respiratory capacity***	*70%*	*2,5*	*0*	*3*
***Autocorrection 3D***	*70%*	*1*	*1*	*3*
***Respiratory education***	*60%*	*1,5*	*0*	*3*
***Equilibrium***	*55%*	*2*	*1*	*3*
***Muscular strength***	*55%*	*2*	*1*	*3*
***Autoelongation***	*50%*	*3*	*1*	*3*
***Increase of ROM***	*50%*	*3*	*1*	*3*
***Neuromotorial control ***	*50%*	*1*	*1*	*3*
***Side-shift***	*50%*	*2*	*1*	*3*
***Stabilisation***	*50%*	*1*	*1*	*4*
***Muscular endurance***	*45%*	*2*	*1*	*3*
***Coordination***	*40%*	*2*	*1*	*3*
***Ergonomy***	*35%*	*2*	*2*	*3*
***General motor capacity***	*25%*	*2*	*1*	*2*

*Psychological aspects*	*10%*	*1*	*1*	*1*
*Scoliosis exercises in groups*	*5%*	*1*	*0*	*0*
*Restoring of physiological spinal curvatures (sagittal plane)*	*5%*	*1*	*1*	*1*
*Correction of contractures and muscles shortening*	*5%*	*1*	*1*	*1*
*Proprioception and tactile*	*5%*	*3*	*3*	*3*
*Neurodynamics*	*5%*	*3*	*3*	*3*
*Theoretical information for the patient and family*	*5%*	*3*	*3*	*3*
*Activities of daily living*	*5%*	*1*	*1*	*1*
*Self perception*	*5%*	*3*	*3*	*3*
*In – brace exercises*	*5%*	*3*	*3*	*3*

**Table 2:** Postmeeting answers. Preferences relate to number of people who chose the single answer, while percentages relate only to people who had a preference to each single aim

Answers: 30	**Preferences**	**Priorities**		
	**%**	**1**	**2**	**3**

*Autocorrection 3D*	97%	90%	0%	7%
*Theoretical information for the patient and family*	87%	53%	27%	7%
*Stabilisation*	87%	50%	23%	13%
*Self perception*	87%	43%	33%	10%
*Activities of daily living*	83%	53%	20%	10%
*Muscular endurance*	83%	30%	33%	20%
*Psychological aspects*	77%	43%	20%	13%
*Respiratory education*	77%	27%	27%	23%
*Neuromotorial control of the spine*	70%	33%	30%	7%
*Proprioception and tactile*	70%	27%	33%	10%
*Equilibrium*	70%	20%	37%	13%
*Restoring of physiological spinal curvatures (sagittal plane)*	67%	57%	7%	3%
*Respiratory capacity*	67%	17%	23%	27%
*Ergonomy*	67%	10%	30%	27%
*Correction of contractures and muscles shortening*	63%	23%	30%	10%
*Scoliosis exercises in groups*	63%	10%	33%	20%
*Renge of Motion*	63%	7%	30%	27%
*Coordination*	60%	13%	37%	10%
*General motor capacity*	60%	3%	37%	20%
*Muscular strength*	57%	10%	27%	20%
*Side-shift*	53%	23%	23%	7%
*Autoelongation*	53%	13%	27%	13%
*In – brace exercises*	50%	10%	23%	17%
*Neurodynamics*	50%	7%	33%	10%

## 3. Results

### 3.1. Premeeting-test (before the consensus meeting)

The results are summarized in Table 1. Data were incomplete for some responders and statistical treatment was not attempted.

#### 3.1.1. Topics with general consensus

The therapeutic aim rated highly important (Median 1) was 3D autocorrection having a high degree of consensus (17/20).

#### 3.1.2. Topics with some consensus

Topics with some consensus were respiratory capacity (14/20) and respiratory education (12/20) (Median 1,5 – 2,5 = medium priority); equilibrium (Median 2 = medium priority; 11/20), muscular strength (Median 2 = medium priority; 11/20), neuromotorical control (Median 1 = high priority; 10/20) and stabilisation (Median 1 = high priority; 10/20).

The other aims given were not rated with a high priority. Nevertheless some consensus was found, with at least 5/20, for instance, considering an improvement of general motor capacity necessary.

#### 3.1.3. Topics with no consensus

Aims added by certain authors included the following:

#### Exercise in groups

Exercises in groups is performed during Scoliosis In-patient Rehabilitation (SIR) in Germany and Barcelone, but also in Switzerland and Israel. The positive psychological impact on scoliosis patients who are rather alone with their deformity helps to cope with the disorder [81-83].

#### Restoration of sagittal profile

This is an integral component '3D Autocorrection' [82].

#### Psychology

Psychological aspects play a key role in physiotherapy. The question is which methodology to be taken and whether PT's are the right professionals trained also in psychological direction. Anyway we have a good psychological impact from group sessions and therefore the question is whether to make specific group training a standard procedure in physical therapy of scoliosis [83].

#### Correction of contractures

When there are any, surely is of importance when contractures inhibit 3D correction, however not with high priority.

#### Proprioception

The use of proprioception, tactile stimulation to improve neurodynamics and self perception is important and is an integral part of many treatment programs to facilitate 3D correction [82,83].

#### Patient and family education

Theoretical information for the patient and family is very important and should be given by physiotherapists as well as by the guiding physician. Generally, training of PT's in theory of scoliosis rehabilitation also is necessary [83].

#### Activities of daily living (ADL)

ADS is performed as a specialized module of treatment during SIR [81,83,86]. But this aim to address ADL should also follow a standardized methodology, perhaps as an addition to the aim to improve ergonomy.

#### Exercise and brace treatment

In brace-treated patients, exercises are not performed regularily in the centre of the senior author but their proposed importance, and the evidence to support this, should be discussed.

### Awareness of the deformation

This important issue, also classified at 'self perception' is integrated into the Lyonaise Method [84,85] and the Schroth programme [82,83,86] as a component of diagnosis and education, as well as a baseline for 'before and after' evaluation.

### 3.2.Postmeeting-questionaire (after the consensus meeting)

The results of the postmeeting-test changed slightly compared to the premeeting values (Table 2). The choice for highest priority for treatment aims were:

*Autocorrection in 3D *(97%),

*Restoration of the sagittal alignment *(67%),

*ADL-training *(83%),

*Theoretical information to the patient *(87%),

*Stabilisation *(87%).

## Discussion

In 1941, a committee of the American Orthopaedic Association undertook a study of methods and results of treatment of idiopathic scoliosis, by interviewing clinicians at sixteen clinics in the United States [87]. Case histories of 425 patients, followed for >1 year after treatment, were evaluated. The goal of the study was to 'establish the present status of this condition, and to clarify, in so far as possible, what can be expected from the present methods of treatment.'

At that time, most clinics prescribed a regime of specialized exercises and/or surgery [87]. Short term results obtained with surgery and with exercise were similar, with little or no improvement obtained for most patients. Among 214 patients treated with spinal fusion, significant loss of correction occurred in 92% of patients, and in 30% of cases the curvature was the same or worse after surgery than before. Long term complications were not available but at short-term followup, the results in 69% of treated patients were rated as 'fair' or 'poor.' Among 185 patients treated with exercise at the 16 clinics surveyed, 69% either remained unchanged or increased by 5–15 degrees, 27% increased by ≥20 degrees, and one curve improved by >10 degrees. Questionnaires revealed that 'most men agree that postural improvement can be expected from a regime of exercises, but the curve itself cannot be decreased by this means.'

In the ensuing decades since this study was published, the routine use of exercise for patients in the United States was largely eliminated (e.g., 65–78). Meanwhile, an ongoing global effort to develop effective surgical methods is reflected in >10,000 peer reviewed articles published, in English, since 1950 and listed in Medline and other searches for scholarly articles. Unfortunately, the lack of success with exercise reported in 1941, unlike the failure of surgery, has not led to a corresponding effort to define improved methods for using physical therapy to treat patients with scoliosis: A parallel search of Medline reveals that fewer than 100 articles exploring the use of exercise-based approaches in the treatment of scoliosis in patients, of any age, have been published.

The routine use of exercise has remained central to therapeutic approaches in many countries [88]. To date, however, the body of literature available to patients and clinicians is of limited use [64]. The relatively limited literature in part reflects clinical traditions which have not placed a high priority on publication. Perhaps more important, a diversity of approaches, standards, and languages limits how accessible and interpretable the available information is to colleagues with common interests [64]: Among several hundred reports of clinical outcome published in recent decades (>600), no fewer than ten different languages were used. The establishment of a scientific society dedicated to research into scoliosis rehabilitation, and a venue for rigorous peer review of results from specialists, are critical first steps in defining the role of physical therapy in treatment of scoliosis.

## Conclusion

A foundational meeting of SOSORT, a new international scientific society dedicated to research on scoliosis rehabilitation, met in Milan, Italy in January 2005. Questionnaires, given before and after the meeting, were used to explore current beliefs, approaches, and goals in clinical practice. The responses to the questionnaires show that, in principle, specialists in scoliosis physiotherapy do not disagree and that several features can be regarded, currently, as standard features in the rehabilitation of scoliosis patients. These features include autocorrection in 3D, training in ADL, stabilizing the corrected posture, and patient education. However, due to a lack of common standards and common terminology the meaning of some questions were understood quite differently. A priority for SOSORT will be to foster a common language and therapeutic standards among the international community specializing in conservative scoliosis management. With the establishment of a clinical and conceptual framework for communication and planning, multicenter studies can be designed to measure the short and long-term efficacy of these approaches in maintaining health and function in children diagnosed with scoliosis.

### Author contributions

MR,  TK, TBG, and TM contributed by reviewing, text editing and adding certain  textfiles and references.

## Competing interests

The author(s) declare that they have no competing interests.
